# Effects of chemokine (C-C motif) receptor 2 and 3 antagonists in rat models of hemorrhagic shock

**DOI:** 10.1371/journal.pone.0284472

**Published:** 2023-04-18

**Authors:** McWayne Weche, Anthony J. DeSantis, Michelle Y. McGee, Garrett A. Enten, Xianlong Gao, Matthias Majetschak

**Affiliations:** 1 Department of Surgery, Morsani College of Medicine, University of South Florida, Tampa, Florida, United States of America; 2 Department of Molecular Pharmacology and Physiology, Morsani College of Medicine, University of South Florida, Tampa, Florida, United States of America; Medical College of Georgia, Augusta, UNITED STATES

## Abstract

Systemic concentrations of chemokine CCL2, an agonist at chemokine receptors CCR2/3/5, have been associated with hemodynamic instability after traumatic-hemorrhagic shock. We reported previously that the CCR2 antagonist INCB3284 prevents cardiovascular collapse and reduces fluid requirements after 30min of hemorrhagic shock (HS), whereas the CCR5 antagonist Maraviroc was ineffective. The effects of CCR3 blockade after HS are unknown and information on the therapeutic potential of INCB3284 after longer periods of HS and in HS models in the absence of fluid resuscitation (FR) is lacking. The aims of the present study were to assess the effects of CCR3 blockade with SB328437 and to further define the therapeutic efficacy of INCB3284. In series 1–3, Sprague-Dawley rats were hemorrhaged to a mean arterial blood pressure (MAP) of 30mmHg, followed by FR to MAP of 60mmHg or systolic blood pressure of 90mmHg. Series 1: 30min HS and FR until t = 90min. SB328437 at t = 30min dose-dependently reduced fluid requirements by >60%. Series 2: 60min HS and FR until t = 300min. INCB3284 and SB328437 at t = 60min reduced fluid requirements by more than 65% (p<0.05 vs. vehicle) and 25% (p>0.05 vs. vehicle), respectively, until t = 220min. Thereafter, all animals developed a steep increase in fluid requirements. Median survival time was 290min with SB328437 and >300min after vehicle and INCB3284 treatment (p<0.05). Series 3: HS/FR as in series 2. INCB3284 at t = 60min and t = 200min reduced fluid requirements by 75% until t = 300min (p<0.05 vs. vehicle). Mortality was 70% with vehicle and zero with INCB3284 treatment (p<0.05). Series 4: INCB3284 and SB328437 did not affect survival time in a lethal HS model without FR. Our findings further support the assumption that blockade of the major CCL2 receptor CCR2 is a promising approach to improve FR after HS and document that the dosing of INCB3284 can be optimized.

## Introduction

Traumatic injury remains a leading cause of death worldwide and hemorrhagic shock is the major cause of potentially preventable death after injuries [1]. While intravenous prehospital fluid resuscitation is important when blood components are not available, it bears well-recognized risks, such as coagulopathy or fluid overload [2]. Despite the urgent need to improve fluid resuscitation strategies, pharmacological interventions that reduce fluid requirements, stabilize hemodynamics and lack significant vasopressor activity are not available.

Various chemokines have been identified as key drivers that initiate and amplify the very early inflammatory response to traumatic-hemorrhagic shock and fluid resuscitation, and systemic concentrations of chemokine (C-C motif) ligand 2 (CCL2), an agonist at chemokine (C-C motif) receptor 2 (CCR2), CCR3 and CCR5, have been associated with hypotension and survival in trauma patients [3–9].

Recently, we reported that blockade of CCR2 with INCB3284 during fluid resuscitation after 30 min of hemorrhagic shock prevents from hemodynamic decompensation and reduces fluid requirements in controlled hemorrhagic shock rat models [10]. In these studies, we also observed that blockade of CCR5 with the Federal Drug Administration approved antagonist Maraviroc did not affect hemodynamics and fluid requirements [10]. The effects of CCR3 blockade on fluid requirements and hemodynamics during resuscitation after hemorrhagic shock, however, have not been evaluated. Moreover, information on the therapeutic potential of INCB3284 after hemorrhagic shock is limited to a single animal model. Thus, the aims of the present study were to evaluate the effects of CCR3 blockade utilizing the selective CCR3 antagonist SB328437 [11] and to further define the therapeutic potential of INCB3284 after longer periods of hemorrhagic shock in a model mimicking pre-hospital crystalloid resuscitation and in a lethal hemorrhagic shock model in the absence of fluid resuscitation.

## Materials and methods

### Drugs

INCB3284 and SB328437 were purchased from Tocris, Bio-Techne Corporation (Minneapolis, MN, USA).

### Hemorrhagic shock models

All procedures were performed in accordance with the National Institutes of Health Guidelines for Use of Laboratory Animals and were approved by the Institutional Animal Care and Use Committee of the University of South Florida (IS00008139). The IACUC specifically reviewed and approved the anticipated mortality in the study design. Male Sprague-Dawley rats (300–405 g) were purchased from Envigo (Indianapolis, IN, USA). The hemorrhagic shock models were performed as described previously [10, 12–14]. In brief, anesthesia induction was performed with the animal and isoflurane-soaked gauze placed in a bell jar. After anesthesia induction, the animals were transferred to the operative field and anesthesia was maintained with 2.7% isoflurane administered via nose-cone inhalation with the SomnoSuite small animal anesthesia system (Kent Scientific Corporation, Torrington, CT, USA). At this dose of isoflurane rats did not respond to noxious stimuli but maintained spontaneous respiration. Using a direct cut-down technique, the left femoral artery was isolated and cannulated with a 24-gauge catheter to allow for blood withdrawal, drug administration, fluid resuscitation and hemodynamic monitoring. The time needed for blood withdrawals and for the injection of drugs or resuscitation fluids in 1 mL volumes was less than 30 s per injection, and thus did not interfere with the continuous monitoring of blood pressures. After catheter placement, isoflurane was decreased to 1.5%. Hemodynamics were continuously monitored with the Surgivet invasive blood pressure monitor (Med-Electronics, Beltsville, MD, USA). Blood pressures were recorded at 1–5 min intervals during the hemorrhage and resuscitation periods. We performed 4 subsequent series of experiments. All experiments in each series were performed in alternating order. In all experiments animals were continuously monitored and remained under general anesthesia until euthanasia or death, as defined by asystole or loss of pulse pressure.

**Series 1:** To assess the effects of SB328437 treatment after hemorrhagic shock and fluid resuscitation, and to be able to compare findings with our previous study on the effects of CCR2 and CCR5 inhibition, rats were hemorrhaged to a mean arterial blood pressure (MAP) target of 30 mmHg for a period of 30 minutes. At the end of the hemorrhagic shock period (t = 30 min), animals were injected with either vehicle (1 mL lactated Ringer’s solution (LR), n = 5), 0.25 μmol/kg SB328437 (n = 3) or 1.1 μmol/kg SB328437 (n = 3) in 1 mL LR, followed by fluid resuscitation with 1 mL bolus injections of LR to maintain a systolic blood pressure of 90 mm Hg or an MAP of 60 mm Hg until t = 90 min, as we described [10]. To avoid fluid overload, bolus injections of LR were limited to 1 mL/min. At t = 90 min, surviving animals were euthanized (5% isoflurane inhalation, bilateral pneumothorax).

**Series 2:** Rats were hemorrhaged to a MAP target of 30 mmHg for a period of 60 min, followed by fluid resuscitation until t = 300 min as in Series 1. At the end of the hemorrhagic shock period (t = 60 min), animals were injected with 1 mL of normal saline (NS) (n = 6), 5 μmol/kg (n = 6) INCB3284 in 1mL NS or 1.1 μmol/kg SB 328437 (n = 6) in 1 mL NS at t = 60 min. At t = 0 min, 60 min, 120 min, 180 min, 240 min and 300 min, blood samples (0.3 ml) were obtained and used for arterial blood gas analyses and measurements of laboratory parameters. At t = 300 min, surviving animals were euthanized (5% isoflurane inhalation, bilateral pneumothorax).

**Series 3:** Rats were hemorrhaged to a MAP of less than or equal to 30 mmHg for a period of 60 min, followed by fluid resuscitation until t = 300 min as in Series 2. At the end of the hemorrhagic shock period (t = 60 min) and at t = 200 min, animals were injected with 1 mL of NS (n = 7) or with 5 μmol/kg INCB3284 in 1mL NS (n = 7). At t = 0 min, 60 min, 120 min, 180 min, 240 min and 300 min, blood samples (0.3 ml) were obtained and used for arterial blood gas analyses and measurements of laboratory parameters. At t = 300 min, surviving animals were euthanized (5% isoflurane inhalation, bilateral pneumothorax).

**Series 4:** The purpose of these experiments was to determine whether treatment with the chemokine receptor antagonists increases shock tolerance, blood pressure and survival time without additional fluid resuscitation. As such, death is an intentional endpoint. Earlier endpoints are unable to answer these questions and alternatives are not available. Rats were hemorrhaged 40% total blood volume (TBV) within 10 min. At t = 15 min, animals were injected with vehicle (1 mL NS, n = 3), 5 μmol/kg INCB3284 in 1mL NS (n = 5) or 1.1 μmol/kg SB 328437 in 1 mL NS (n = 5). Animals were then hemorrhaged 2% TBV every 5 minutes until death.

### Arterial blood gases and laboratory parameters

Arterial blood gases, electrolytes, creatinine, lactate, hematocrit and hemoglobin were analyzed in series 2 and 3 in 60 min intervals using the Element point of care veterinary blood gas, electrolyte and critical care analyzer (Cuattro Veterinary USA, Loveland, CO, USA).

### Data analyses and statistics

Data are presented as mean ± standard error (SE). Data were analyzed by 2-way analysis of variance (ANOVA) with Dunnett’s multiple comparisons tests. Survival curves were analyzed using the log-rank test. Proportions were compared with the Fisher’s exact test. All data analyses were calculated with the GraphPad Prism program (GraphPad Software Version 9.3.1 (350), December 7, 2021). A two-tailed p<0.05 was considered significant.

## Results and discussion

### Series 1: Single dose SB328437 treatment reduces fluid requirements within the first hour after a 30 min hemorrhagic shock period

**Fig 1:**
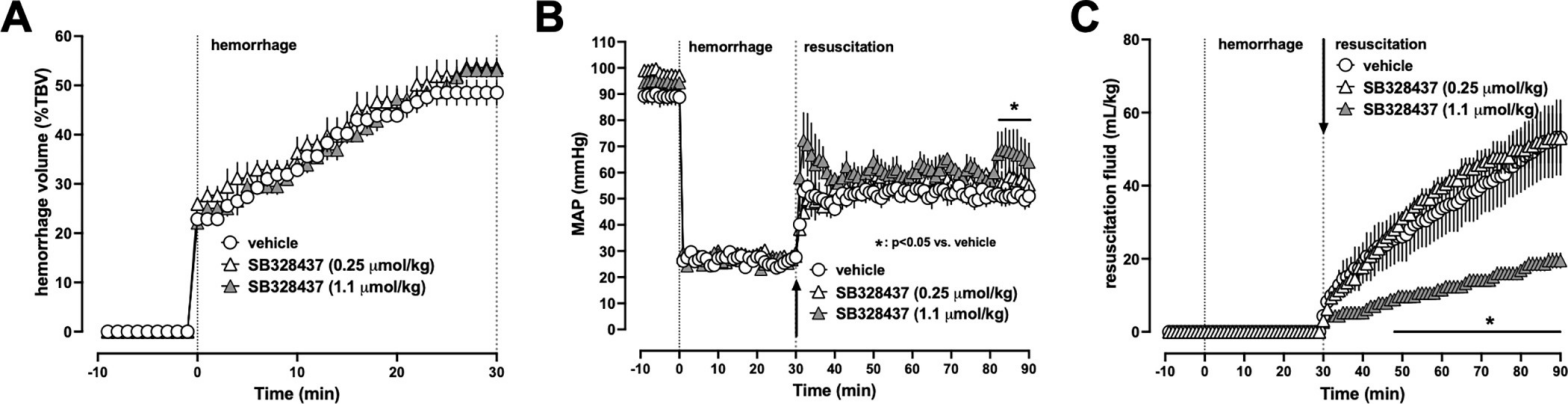
Single dose SB328437 treatment reduces fluid resuscitation requirements after 30 min of hemorrhagic shock. All data are mean ± SE. Arrows represent the time point of drug/vehicle injection. Open circles: animals treated with vehicle (n = 5). Open triangles: animals treated with 0.25 μmol/kg SB328437 (n = 3). Grey triangles: animals treated with 1.1 μmol/kg SB328437 (n = 3). *: p < 0.05 vs. animals treated with vehicle. (A) Hemorrhage volumes for maintain mean arterial blood pressure (MAP of 30 mmHg). %TBV: percent of total blood volume. (B) MAP (mmHg) (C) Fluid resuscitation in mL/kg required to maintain MAP of 60 mmHg or systolic blood pressure (SBP) of 90 mmHg.

To assess whether blockade of CCR3 affects hemodynamics and fluid requirements after hemorrhagic shock, we employed the same animal model that we previously utilized to characterize effects of the CCR2 and CCR5 antagonists INCB3284 and Maraviroc, respectively [10]. Due to the limited solubility of SB328437, the highest dose of SB328437 that could be administered was 1.1 μmol/kg. There were no differences in any physiological parameters among groups at baseline. The hemorrhage volumes to achieve the target MAP during the shock period were comparable among the groups (Fig 1A). MAPs between groups were indistinguishable during the shock period and all animals could be resuscitated to the target MAP and survived the observation period (Fig 1B). While MAP was indistinguishable between vehicle-treated animals and animals treated with 0.25 μmol/kg SB328437, MAP at the end of the resuscitation period (t = 80–90 min) was higher in animals treated with 1.1 μmol/kg SB328437 (Fig 1B). Fluid requirements to achieve the MAP target during the resuscitation period were comparable in vehicle-treated animals and in animals treated with 0.25 μmol/kg of SB328437 (Fig 1C). As compared with vehicle-treated animals, fluid requirements were reduced by 63 ± 4% in animals treated with 1.1 μmol/kg SB328437 (fluid requirements (mean ± SE): vehicle– 53 ± 10 mL/kg; 0.25 μmol/kg SB328437–53 ± 7 mL/kg; 1.1 μmol/kg SB328437–19.5 ± 2 mL/kg, p<0.05 vs. vehicle, Fig 1C). These observations suggest that blockade of CCR3 with SB328437 dose-dependently reduces fluid requirements in short term resuscitation experiments. The fluid sparing effects of 1.1 μmol/kg SB328437 are comparable with the effects of 5 μmol/kg INCB3284 that we observed in the same model previously [10].

### Series 2: Single dose INCB3284 treatment, but not SB328437 treatment, transiently reduces fluid requirements after a 60 min hemorrhagic shock period

**Fig 2:**
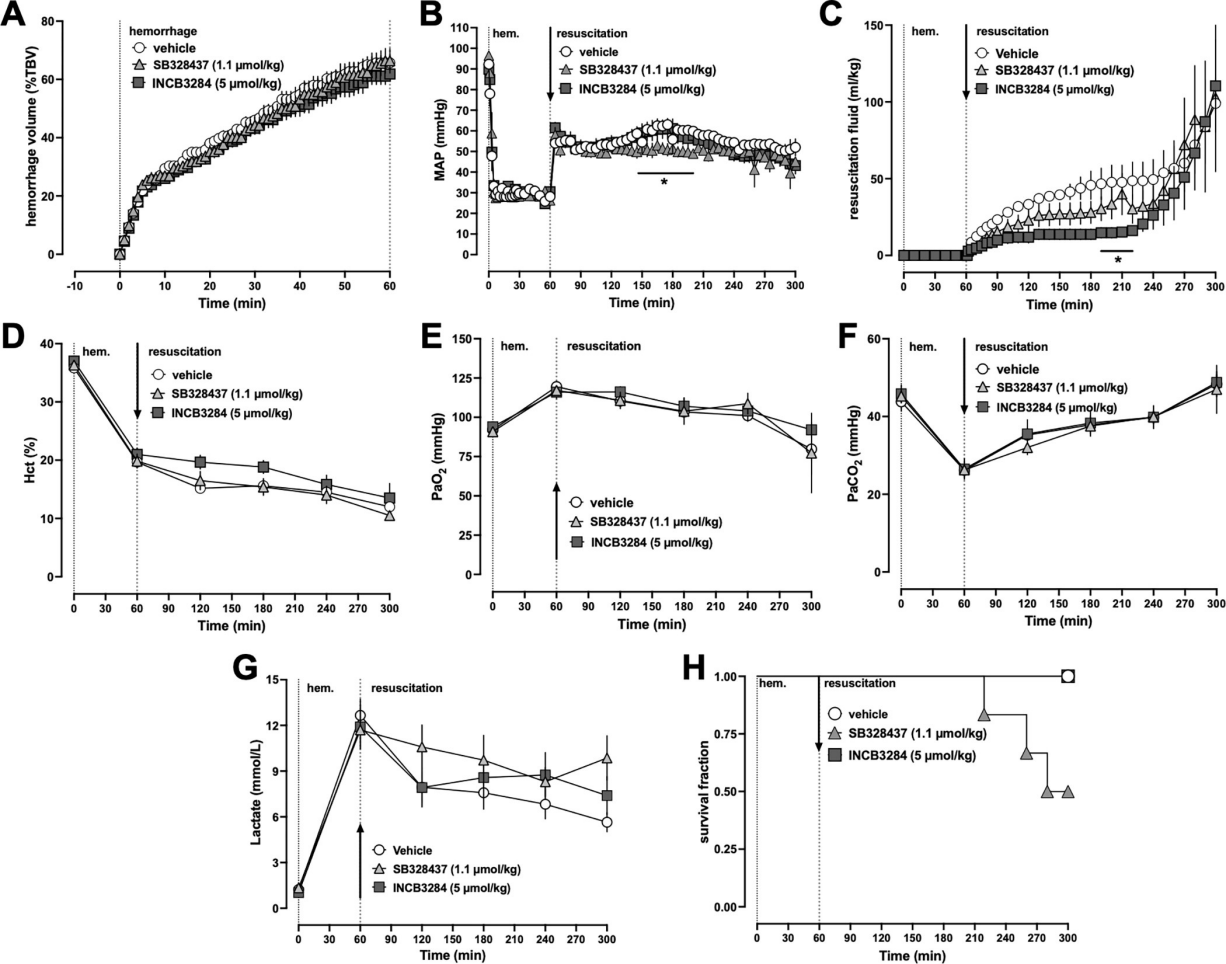
Single dose INCB3284 treatment transiently reduces fluid resuscitation requirements after 60 min of hemorrhagic shock. All data are mean ± SE. Arrows represent the time point of drug/vehicle injection. Open circles: animals treated with vehicle (n = 6). Grey triangles: animals treated with 1.1 μmol/kg SB328437 (n = 6). Grey squares: animals treated with 5 μmol/kg INCB3284 (n = 6). *: p < 0.05 vs. animals treated with vehicle. (A) Hemorrhage volumes for maintain mean arterial blood pressure (MAP of 30 mmHg). %TBV: percent of total blood volume. (B) MAP (mmHg) (C) Fluid resuscitation in mL/kg required to maintain MAP of 60 mmHg or systolic blood pressure (SBP) of 90 mmHg. (D) Hct%: Hematocrit values in %. (E) PaO_2_: Partial pressure of oxygen in arterial blood. (F) PaCO_2_: Partial pressure of carbon dioxide in arterial blood. (G) Plasma lactate concentration (mmol/L). (H) Kaplan-Meier survival curve.

To assess therapeutic efficacy of SB328437 and INCB3284 in a more severe model of hemorrhagic shock and during clinically more relevant periods of fluid resuscitation, we treated animals with vehicle, 1.1 μmol/kg SB328437 or 5 μmol/kg INCB3284 after 60 min of hemorrhagic shock and performed fluid resuscitation until t = 300 min. As in series 1, there were no differences among groups at baseline and the hemorrhage volumes to achieve the target MAP of 30 mmHg were indistinguishable (Fig 2A). MAPs during the shock period (Fig 2B) and lactate concentrations at the end of the shock period (Fig 2G) were indistinguishable between groups, suggesting comparable degrees of shock severity. When compared with vehicle treatment, treatment with INCB3284 reduced fluid resuscitation requirements to achieve the target MAP by 65% until t = 220 min (p<0.05 vs. vehicle, Fig 2B/2C). With SB328437 treatment, however, MAP during fluid resuscitation was lower between t = 140–200 min and fluid requirements could not be significantly reduced (Fig 2B/2C). After t = 220 min, all animals developed a steep increase in fluid requirements, suggesting hemodynamic decompensation. Hematocrit values (Fig 2D) and blood gases (Fig 2E/2F) were comparable among the groups. Because lactate concentrations during the resuscitation period are affected by LR infusion, absolute concentrations should be interpreted with caution. Nevertheless, there were no differences in lactate concentrations during the resuscitation period between groups (Fig 2G). Furthermore, only 3 of 6 animals that were treated with SB328437 survived the resuscitation period, whereas all vehicle and INCB3284-treated animals survived (Fig 2F). Median survival time with SB328437 treatment was 290 min and undefined with vehicle and INCB3284 treatment (p < 0.05 vs. SB328437-treatment). It should be noted, however, that the number of animals in each group was small and survival proportions at t = 300 min were not significantly different between SB328497 and vehicle treated animals (p = 0.18). Because hematocrit values, blood gases and lactate levels were comparable among groups during the entire observation period and fluid requirements were indistinguishable after t = 220 min, we cannot exclude that some animals after vehicle or INCB3284 treatment would have died shortly after the end of the resuscitation period and that the median survival time is close to 300 min in all animals. Irrespective of a potential survival disadvantage, however, SB328437 treatment after 60 min of hemorrhagic shock lost the fluid sparing effect that we observed in series 1 after 30 min of hemorrhagic shock. In contrast, INCB3284 treatment transiently reduced fluid requirements until t = 220 min after 60 min of hemorrhagic shock in the present study, suggesting that the therapeutic potential of INCB3284 to reduce fluid resuscitation requirements is higher than of SB328437.

### Series 3: Redosing of INCB3284 reduces fluid requirements and prevents hemodynamic decompensation after a 60 min hemorrhagic shock period

**Fig 3:**
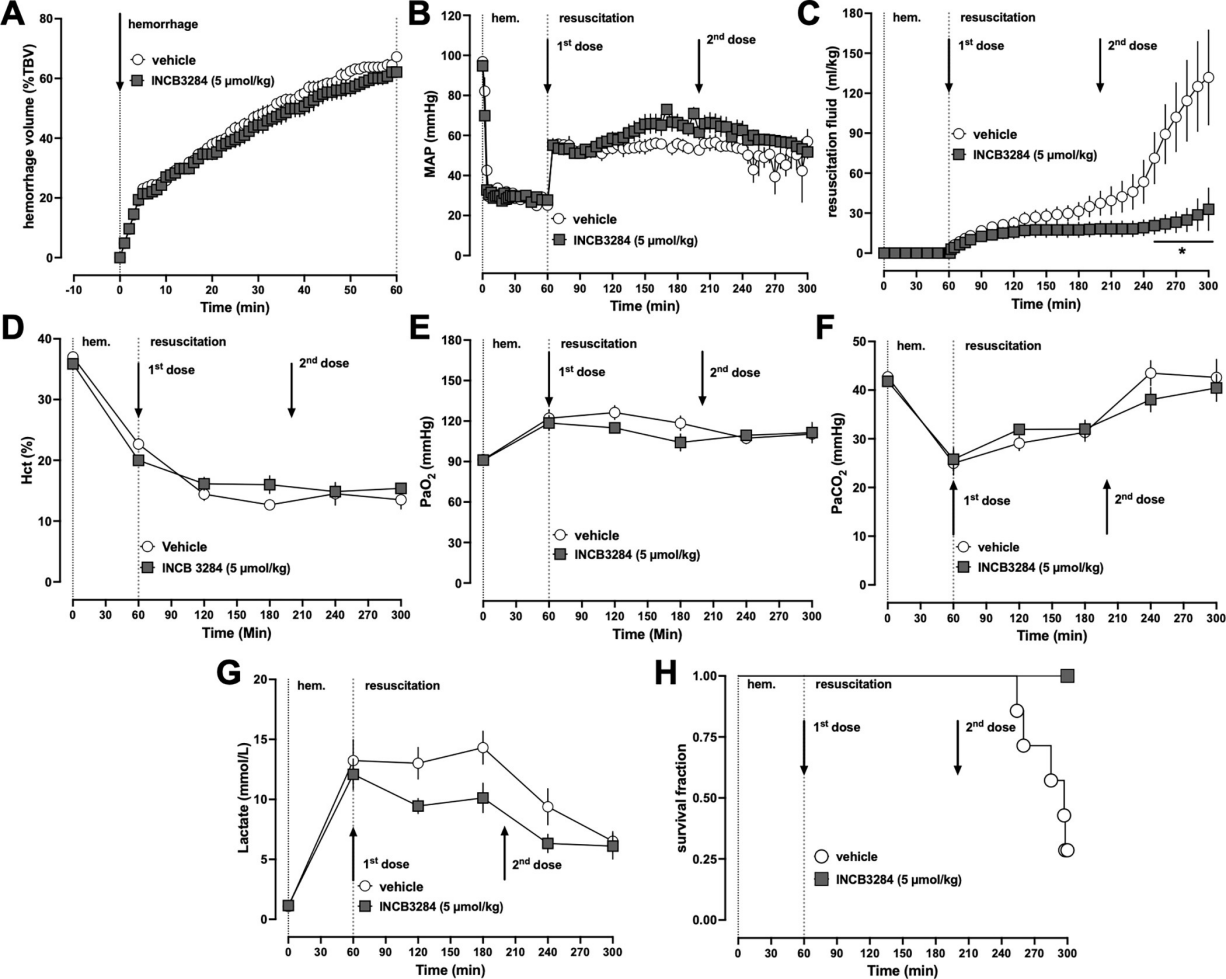
Redosing of INCB3284 reduces fluid resuscitation requirements after 60 min of hemorrhagic shock. All data are mean ± SE. Arrows represent the time points of drug/vehicle injection. Open circles: animals treated with vehicle (n = 7). Grey squares: animals treated with 5 μmol/kg INCB3284 (n = 7). *: p < 0.05 vs. animals treated with vehicle. (A) Hemorrhage volumes for maintain mean arterial blood pressure (MAP of 30 mmHg). %TBV: percent of total blood volume. (B) MAP (mmHg) (C) Fluid resuscitation in mL/kg required to maintain MAP of 60 mmHg or systolic blood pressure (SBP) of 90 mmHg. (D) Hct%: Hematocrit values in %. (E) PaO_2_: Partial pressure of oxygen in arterial blood. (F) PaCO_2_: Partial pressure of carbon dioxide in arterial blood. (G) Plasma lactate concentration (mmol/L). (H) Kaplan-Meier survival curve.

The half-life of INCB3284 has been reported to be 168 min in rats [15]. In combination with the observations that 5 μmol/kg INCB3284 reduced fluid requirements after hemorrhagic shock, whereas a dose of 1.1 μmol/kg INCB3284 was ineffective [10], it appeared possible that systemic concentrations of INCB3284 after t = 200–220 min declined below the threshold of therapeutic efficacy. To test whether redosing of INCB3284 improves therapeutic efficacy, we utilized the same model as in series 2 and administered vehicle or INCB3284 at the beginning of fluid resuscitation (t = 60 min) and at t = 200 min. Similar to series 1 and 2, there were no differences among groups at baseline, the hemorrhage volumes to achieve MAP of 30 mmHg (Fig 3A) and MAPs during the shock period (Fig 3B) were indistinguishable. As expected based on our findings in series 2, fluid requirements to maintain target MAP during resuscitation increased rapidly after t = 220 min and reached 131 ± 35 mL/kg at t = 300 min after vehicle treatment (Fig 3B/3C). With double dosing of INCB3284, the steep increase in fluid requirements was prevented and total fluid requirements were reduced by 75% (33 ± 16 mL/kg, p<0.05 vs. vehicle treatment), when compared with vehicle treated animals (Fig 3B/3C). As in series 2, hematocrit values, blood gases and lactate concentrations at the end of the hemorrhage period and during the resuscitation period were not significantly different among groups (Fig 3D–3G). Unlike in series 2, mortality was 71% and median survival time 297 min after vehicle treatment (Fig 3H). With double dosing of INCB3284, however, mortality was reduced to zero (p<0.05 vs. vehicle treatment) and median survival time undefined (p<0.05 vs. vehicle treatment, Fig 3H).

Given that fluid requirements and other physiological parameters of vehicle treated animals were comparable in series 2 and 3, our observations suggest that the lack of mortality after vehicle treatment in series 2 was a chance observation caused by a small sample size, which argues against a survival disadvantage with single dose SB328437 treatment. The combined mortality in vehicle-treated animals from series 2 and 3 (n = 13 animals) was 38.5% and median survival time remains undefined, which is not significantly different from zero mortality that we observed with double dosing of INCB3284. This indicates that larger cohort sizes are required to clarify whether double dosing of INCB3284 provides a relevant survival benefit. Nevertheless, our finding that double dosing of INCB3284 prevented the steep increase in fluid requirements that was detectable in all vehicle treated animals in series 2 and 3 and in animals after single dose treatment with INCB3284 in series 2, demonstrates that dosing of INCB3284 can be optimized to increase its fluid sparing effects during resuscitation from hemorrhagic shock and suggests that treatment of animals with an optimized dosing regimen of INCB3284 also prevents, or at least delays, hemodynamic decompensation in a more severe model of hemorrhagic shock.

### Series 4: Treatment with INCB3284 and SB328437 does not increase shock tolerance

**Fig 4:**
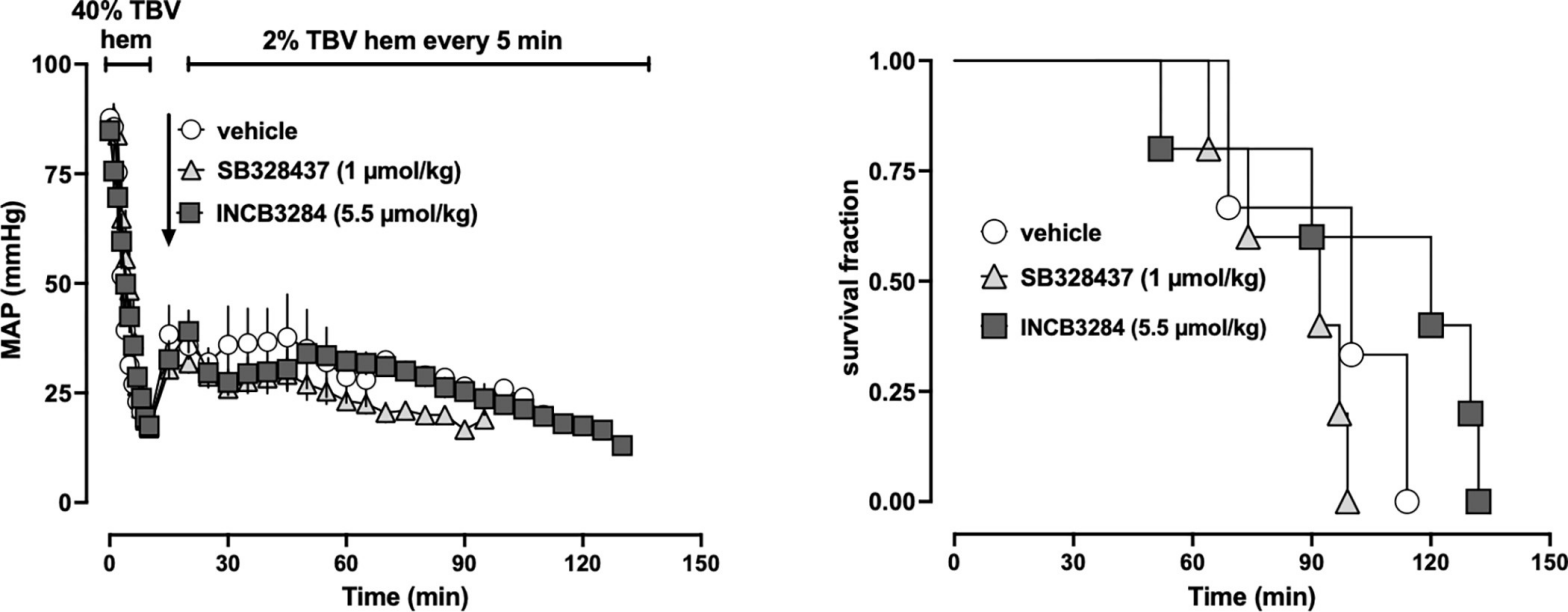
SB328437 and INCB3284 do not affect survival during lethal hemorrhage without fluid resuscitation. All data are mean ± SE. Arrows represent the time point of drug/vehicle injection. Open circles: animals treated with vehicle (n = 3). Grey triangles: animals treated with 1.1 μmol/kg SB328437 (n = 5). Grey squares: animals treated with 5 μmol/kg INCB3284 (n = 5). (A) MAP (mmHg). (B) Kaplan-Meier survival curve.

To assess whether INCB3284 or SB328437 may increase tolerance to severe hemorrhagic shock, we utilized a model designed to mimic continuous bleeding in the absence of fluid resuscitation. As shown in Fig 4A, when animals were treated with vehicle, 5 μmol/kg INCB3284 or 1 μmol/kg SB328437 after 40% TBV hemorrhage, MAP was indistinguishable among groups during subsequent 2% TBV hemorrhages in 5 min intervals. Median survival times were not significantly different between groups (median survival time: vehicle treatment– 72 min; SB328437–64 min; INCB328437–80 min, Fig 4B).

## Conclusions

Taken together, our observations suggest that blockade of CCR3 during resuscitation results in fluid sparing effects that depend on the duration of the hemorrhagic shock period and are lost when the duration of the shock period is extended. Moreover, our findings on the effects of INCB3284 further support the assumption that blockade of CCR2 is a promising pharmacological approach to reduce fluid requirements and to prevent or delay hemodynamic decompensation even after prolonged periods of hemorrhagic shock. Our observations document that the therapeutic efficacy of the CCR2 antagonist INCB3284 can be optimized and prolonged by an improved dosing regimen. This study is limited in scope by a relatively small sample size in each series of experiments and by a maximal resuscitation period of 4 hours. As such, it remains to be determined whether any of the drugs affect survival after longer time periods and whether the fluid sparing effects of INCB3284 can be maintained over extended time periods by subsequent redosing or by continuous drug infusion. Although we did not observe adverse effects of INCB3284 and SB328437, we performed only a limited physiological assessment with measurements of basic laboratory parameters. In addition, our study was designed as an efficacy testing to evaluate fluid sparing effects during resuscitation from hemorrhagic shock. As such, this study does not provide information on physiological and molecular mechanisms by which CCR3 and CCR2 blockade affect resuscitation fluid requirements and on the possible side effect profile of the drugs. Based on previous observations indicating that CCL2 and CCR2 are involved in the regulation of vascular function, however, it appears likely that direct effects of CCR2 blockade on endothelial or vascular smooth muscle function mediate the observed effects [16–18]. We believe that the profound fluid sparing effects of INCB3284 that we observed in the present study further justify its detailed pharmacological characterization in small and large animal models, and the assessment of its side effect profile as a drug candidate to improve fluid resuscitation from traumatic-hemorrhagic shock.
